# Genotype distribution and treatment response among incarcerated drug-dependent patients with chronic hepatitis C infection

**DOI:** 10.1371/journal.pone.0191799

**Published:** 2018-02-01

**Authors:** Chun-Han Cheng, Ching-Chung Lin, Huan-Lin Chen, I-Tsung Lin, Chia-Hsien Wu, Yuan-Kai Lee, Ming-Jong Bair

**Affiliations:** 1 Division of Gastroenterology, Department of Internal Medicine, Taitung Mackay Memorial Hospital, Taitung City, Taiwan; 2 Mackay Medical College, New Taipei, Taiwan; 3 Division of Gastroenterology, Department of Internal Medicine, Mackay Memorial Hospital, Taipei, Taiwan; National Taiwan University Hospital, TAIWAN

## Abstract

The prevalence of hepatitis C virus (HCV) infection is disproportionately high among prisoners, especially among those who are drug-dependent. However, current screening and treatment recommendations are inconsistent for this population, and appropriate care is not reliably provided. To address these problems, the present study aimed to identify unique characteristics and clinical manifestations of incarcerated patients with HCV infection. We included incarcerated patients who received treatment with pegylated-interferon combined with ribavirin at Mackay Memorial Hospital in Taitung and were serving sentences at either the Taiyuan Skill Training Institute or the Yanwan Training Institute. HCV genotypes 1 (41.4%), 3 (25.9%), and 6 (24.1%) were the most prevalent in the incarcerated patients. During the study period, we analyzed treatment response among 58 incarcerated patients and compared obtained results with treatment response among 52 patients who were living in the community. Higher sustained virological response rate was observed among patients with incarceration and HCV genotype other than 1. The odds ratios (corresponding 95% confidence intervals) for incarceration and genotype 1 were 2.75 (1.06–7.11) and 0.37 (0.14–0.99), respectively. Better treatment compliance among incarcerated patients might partially explain these results. The results of this study suggest that treatment of prisoners with HCV infection is feasible and effective. More appropriate and timely methods are needed to prevent HCV transmission among injection drug users inside prisons.

## Introduction

Hepatitis C virus (HCV) infection is a global health problem that can lead to chronic hepatitis, cirrhosis, and hepatocellular carcinoma. The prevalence of HCV infection is high among prisoners [1–4]. Transmission of HCV within prisons may occur via tattooing, sharing of injecting apparatus, and other activities involving potential blood contact, such as barbering and fighting [5, 6]. Injecting drug users (IDUs) are at a particularly high risk of HCV transmission [7, 8]. Given the high prevalence of HCV infection, it is important to identify and evaluate prisoners for antiviral treatment. In addition, prisons could serve as a reservoir of amplification of HCV transmission in the community after prisoners’ release; this fact highlights the importance of early HCV eradication in the correctional system [9]. Unfortunately, in most countries, prisoners have fewer opportunities to receive medical assistance than other citizens [10]. Thus, a health burden of liver-related morbidities might exist in patients within correctional systems. Appropriate management of HCV infection among prisoners may provide a significant public health improvement.

HCV has six major genotypes and multiple subtypes. The distribution of HCV genotypes varies geographically. In Taiwan, the most prevalent genotype is 1b, followed by 2a and 2b, with genotypes 3 and 6 being very rare in the general population [11, 12]. The mode of viral transmission may influence the predominance of certain genotypes. A higher prevalence of genotypes 3 and 6 has been reported in IDUs [7, 13–15]. However, relatively few studies have been conducted on HCV infection in prison populations. Correctional systems continue to face challenges in developing programs to screen, diagnose, and treat HCV infection in their incarcerated populations.

Factors that have been used to predict the efficacy of pegylated-interferon (peg-IFN) combined with ribavirin (RBV) therapy include HCV genotype, baseline hepatitis C viral load, and host characteristics (e.g., sex, age, hepatic fibrosis, innate and adaptive immunity, and various genetic factors). Treatment response among incarcerated patients with HCV infection remains unclear. The correctional system may possess unique capabilities and an appropriate environment for treatment of HCV infection. However, previous reports focusing on therapeutic response have revealed controversial results [8, 16–20]. Recommendations for general screening and diagnostic and therapeutic approaches for HCV infection in prisoners have been issued by only a few experts [21, 22] and often lack consistency.

Taitung Mackay Memorial Hospital has treated prisoners with HCV infection since January 2014 in accordance with the policies of the National Health Insurance system. These patients were incarcerated in the Taiyuan Skill Training Institute and the Yanwan Training Institute. The distinguishing features of these patients are that they were all men and were all drug dependent. The aims of this study were to elucidate the characteristics and clinical manifestations of patients with HCV infection from this specific population.

## Materials and methods

### Study population

We enrolled all incarcerated patients with chronic HCV infection who started to receive antiviral therapy between January 2014 and June 2016 at Taitung Mackay Memorial Hospital. The inclusion criteria were patients aged older than 20 years with chronic HCV infection, defined as detectable anti-HCV antibody and serum HCV ribonucleic acid (RNA) for ≥ 6 months. They were treated with peg-IFN combined with RBV according to the National Health Insurance clinical practice guidelines. All of them were drug dependent, incarcerated in either the Taiyuan Skill Training Institute or the Yanwan Training Institute, and received their antiviral courses while incarcerated. Patients were excluded if they had the following comorbidities: autoimmune disorders, cytopenia, psychiatric disorders, thyroid diseases, decompensated cirrhosis, or hepatic failure.

At the beginning of treatment, all patients had received therapeutic education with a reference nurse for the treatment course, possible side effects, and management. All patients were treated with peg-IFN-α-2a (180 μg once per week). Patients with HCV genotypes 1 and 6 received weight-based RBV (1000 mg/day if their weight was < 75 kg or 1200 mg/day if their weight was ≥ 75 kg). Patients with HCV genotypes 2 and 3 received a fixed dose of 800 mg/day RBV. The sustained virological response (SVR) was defined as serum HCV RNA being undetectable through 24 weeks after the cessation of treatment. Rapid virological response (RVR) was defined as undetectable HCV RNA at Week 4 of treatment. Treatment conformed to National Health Insurance guidelines. The complete treatment duration was 24 weeks if patients achieved RVR; if not, treatment was prolonged to 48 weeks.

In the present study, we compared baseline characters and treatment response between patients who were incarcerated and those who were living in the community. We enrolled all community patients who started to receive peg-IFN combined with RBV therapy for HCV infection at the same hospital during the same period as the incarcerated patients. The same inclusion and exclusion criteria as for incarcerated patient group were applied. However, only male patients were included, because the prisoners examined in this study were all men. A total of 58 incarcerated and 52 community patients with HCV infection were analyzed to determine treatment response.

This was an observational study. All included patients received similar medical care to other patients with HCV infection. This study was conducted in accordance with the Declaration of Helsinki and was approved by the Institutional Review Board of MacKay Memorial Hospital (Number 17MMHIS026).

### Assessment

The following baseline information was collected: patient age, concurrent infections with chronic hepatitis B virus (HBV) and human immunodeficiency virus (HIV), HCV genotype, HCV RNA level, aspartate aminotransferase (AST) level, alanine aminotransferase (ALT) level, total bilirubin level, white blood cell count, hemoglobin level, platelet level, and renal function.

HCV genotyping was performed using primer-specific polymerase chain reaction (PCR) and direct PCR population sequencing with an ABI 3730 sequencer. Serum HCV RNA viral loads were quantified using a Roche Amplicor PCR assay, for which the lowest level of detection was 15 IU/mL. Patients whose HCV RNA levels were not measured at the time point of SVR and patients who did not complete the scheduled course of treatment because of side effects were coded as treatment failures.

### Statistical analysis

Data are presented as numbers (percentages) or means ± standard deviations. The statistical significance of the continuous data was determined using Student’s *t* test, and the significance of the differences between the categorical variables was determined using the Chi-square test. For continuous variables with wide distribution, we presented median (interquartile range) statistics employing Mann-Whitney U test. Multivariate analyses of categorical variables were conducted using binary logistic regression. Statistical analyses were performed using SPSS 19.0 (SPSS Inc., Chicago, IL, USA). All statistical analyses involved testing two-sided hypotheses, and the significance level was defined as *p* < 0.05.

## Results

### Patient characteristics and HCV genotypes distribution pattern

**Fig 1:**
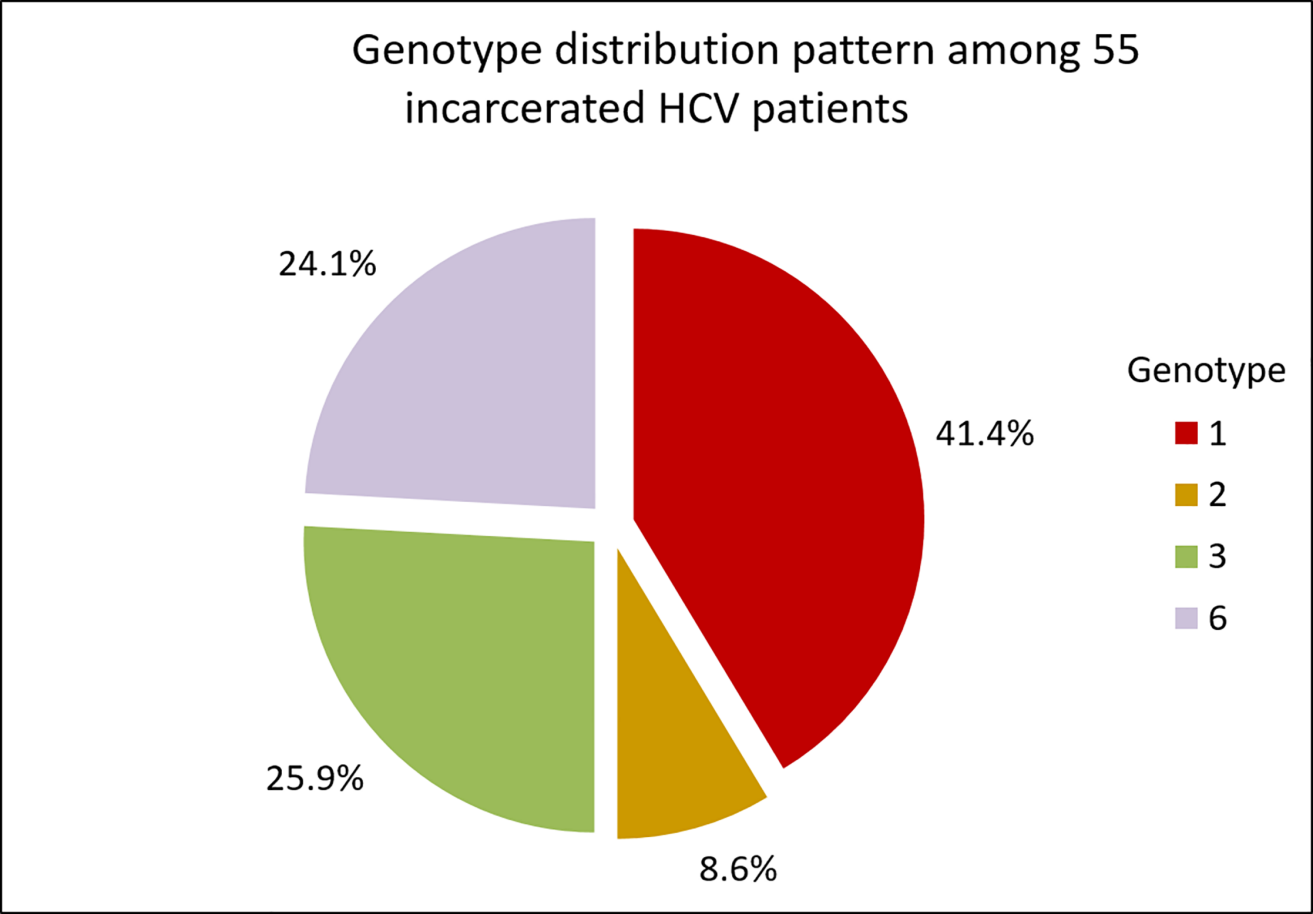
Genotype distribution pattern among 58 incarcerated patients with chronic hepatitis C virus (HCV) infection who received antiviral treatment.

The baseline characteristics of the incarcerated and non-incarcerated male patients with HCV infection in our study are shown in Table 1. The mean age was significant lower among incarcerated patients with HCV infection. All 58 incarcerated patients were men and had a history of drug dependency. In this specific population, nine (15.5%) patients were coinfected with HIV and three (5.2%) with HBV. At the baseline, 75.9% of these patients had HCV viral loads larger than 400,000 IU/mL. The most prevalent genotype was genotype 1 (41.4%), followed by genotypes 3 (25.9%) and 6 (24.1%), as shown in Fig 1.

**Table 1:** Baseline characteristics of incarcerated and community male patients with chronic HCV infection.

	Incarcerated (n = 58)	Non-incarcerated (n = 52)	*p* value
Age (years, mean ± SD)	41.7 ± 8.1	56.5 ± 9.4	< 0.001
Drug abuser	58 (100)	*	*
HBV co-infection	3 (5.2)	5 (9.6)	*
HIV co-infection	9 (15.5)	*	*
HCV genotype 1	24 (41.4)	30 (57.7)	0.09
HCV RNA > 400000 IU/mL	44 (75.9)	33 (63.5)	0.16
^a^AST (IU/L, median (IQR))	53.0 (41.5)	66.5 (45)	0.11
^a^ALT (IU/L, median (IQR))	86.0 (47.0)	95.5 (87.5)	0.45
Total bilirubin (mg/dL, mean ± SD)	1.0 ± 0.3	0.9 ± 0.4	0.10
Creatinine (mg/dL, mean ± SD)	0.9 ± 0.1	0.8 ± 0.2	0.16
Hemoglobin (g/dL, mean ± SD)	14.5 ± 1.4	14.1 ± 1.4	0.07
WBC (10^3^/μL, mean ± SD)	6.0 ± 2.0	5.5 ± 1.8	0.10
^a^Platelet count (10^3^/μL, median (IQR))	187.0 (77.0)	160.0 (77.5)	0.15

Categorical data are presented as number (percentage).

Student’s *t* test was used for continuous data and the Chi-squared test was used for categorical variables.

*In these cells, the information was not collected completely, so we could not perform statistical analysis.

^a^These continuous variables showed wide distribution. They were presented with median (interquartile range) statistics employing Mann-Whitney U test.

ALT = alanine aminotransferase; AST = Aspartate aminotransferase; HBV = hepatitis B virus; HCV = hepatitis C virus; HIV = human immunodeficiency virus; IQR = interquartile range; RNA = ribonucleic acid; SD = standard deviation; WBC = white cell count.

### Treatment response

**Fig 2:**
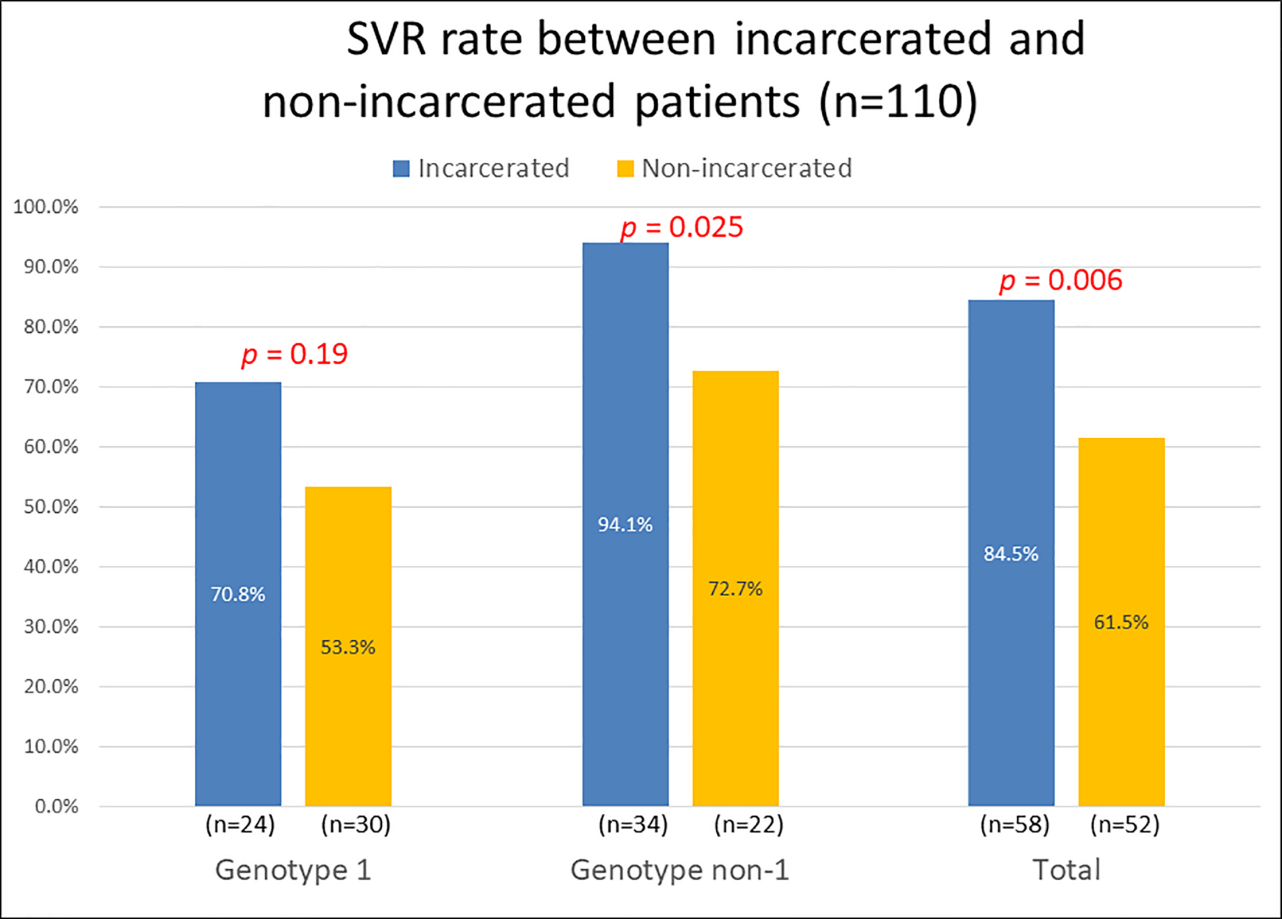
Therapeutic response of incarcerated and community patients with genotype 1 and other genotypes of HCV. The sustained virological response rate was significantly higher in prisoners. However, the difference was not statistically significant in patients with HCV genotype 1.

We analyzed several factors to predict treatment response. The SVR rate was 84.5% among the incarcerated patients and 61.5% among the community patients. Of nine incarcerated patients who did not achieve SVR, two patients showed non-response, one had virological breakthrough, two showed virological relapse, and four patients were lost after treatment completion. Fig 2 displays the treatment response among patients with different HCV genotypes. The SVR rates for genotype 2, 3, and 6 in prisoners were 100.0%, 93.3%, and 92.9%, respectively. In community patients, the SVR rate for genotype 2 and 6 were 73.7% and 66.7%, respectively. No pure HCV genotype 3 was detected in community patients. The statistically difference of SVR rate for genotype 2, 3, and 6 between these two groups could not be obtained because of the small number of included patients. In addition, the compliance was better in prisoners (eight community patients did not receive regular follow-up after completing treatment and four patients discontinued anti-viral therapy because of intolerance of adverse effects). Among the four patients with intolerance because of the adverse effects, two patients complained of headache and myalgia, whereas the other two patients had fever, nausea, and weight loss. Only two incarcerated patients were missed after treatment and no incarcerated patient stopped antiviral therapy because of intolerance.

Our analysis of factors associated with treatment response demonstrated that patients who were incarcerated, infected with HCV genotype non-1, and achieved RVR were significantly associated with SVR (Table 2). Multivariate analysis revealed that incarceration and an HCV genotype other than 1 were independent predictors of successful treatment (Table 3).

**Table 2:** Factors associated with SVR in male HCV-infected patients (n = 110).

	SVR (n = 81)	non-SVR (n = 28)	*p* value
Age (years, mean ± SD)	48.2 ± 11.4	50.1 ± 10.9	0.44
Incarcerated patients	49 (60.5)	9 (31.0)	**0.006**
HCV RNA ≥ 400000 IU/mL	54 (66.7)	23 (79.3)	0.07
HCV genotype 1	33 (40.7)	21 (72.4)	**0.003**
RVR	62 (76.5)	14 (48.3)	**0.005**

Data are presented as numbers (percentages) or means ± standard deviations

Student’s *t* test was used for continuous data and the Chi-squared test was used for categorical variables.

HCV = hepatitis C virus; RNA = ribonucleic acid; RVR = rapid virological response; SVR = sustained virological response.

**Table 3:** Multivariate analysis of predictors for SVR (n = 110).

	Odds ratio	95% CI	*p* value
Incarcerated vs. non-incarcerated	2.75	1.06–7.11	**0.037**
Genotype (1 vs. non-1)	0.37	0.14–0.99	**0.049**
RVR	2.38	0.90–6.29	0.08

Logistic regression was performed.

CI = confidence interval; RVR = rapid virological response; SVR = sustained virological response.

## Discussion

The seroprevalence of HCV infection is higher in prisons [1, 2]. One review article stated that HCV infection prevalence ranges from 3.1% to 38% among prisoners [8]. This may be because of unsafe lifestyles and psychiatric and social problems. In our study, we were unable to evaluate all prisoners at the two facilities; consequently, the true prevalence was not determined. However, when we retrospectively reviewed our cohort, the prisoners accounted for 30–40% of the populations of their facilities. This might support a high prevalence of HCV infection in incarcerated people, because the frequency of general clinical visits was much lower in the prisoners than in the community patients in our study. Another reason for the high number of prisoners receiving antiviral treatment was National Health Insurance policies, which had recently begun to support treatment for prisoners during our study period.

As an HCV endemic area, regional differences of HCV genotype distribution might also exist in Taiwan [11, 23]. In our previous study, the most prevalent genotype in southeastern Taiwan was 1b, followed by 2a [23]. The mode of viral transmission may also influence the predominance of certain genotypes. A higher prevalence of HCV genotype 6 has been reported in IDUs in Taiwan [7, 15]. Some studies have shown a higher frequency of genotype 3 in prisoners [13, 14]. In the general population, genotype 3 is predominant in Thailand, Malaysia, India, and Pakistan [24]. Genotype 6 is restricted to South China, Southeast Asia, and migrant patients from endemic countries [25]. These HCV genotypes may be widely distributed via the routes used for smuggling illegal drugs. In incarceration settings, injection drug use, tattooing, and sexual contact are the main risk factors for HCV infection [8]. In our study, all incarcerated patients had a history of drug dependency, and genotypes 3 and 6 accounted for 25.9% and 24.1%, respectively, of the population. This emphasized the association between the route of HCV transmission and genotype distribution.

The 5.2% and 15.5% coinfection rates for HBV and HIV, respectively, were probably because of similarities in the viral transmission routes. In Taiwan, the prevalence of HCV and HIV coinfection among IDUs is relatively high and has been gradually increasing [26]. The high prevalence of coinfection observed in the present study indicates that drug use is an important risk factor. Therefore, more efforts are required to prevent HCV transmission among IDUs.

Several factors have been shown to influence treatment outcomes in patients with HCV infection, including age, pretreatment HCV RNA levels, HCV genotype, alcohol consumption, liver fibrosis status, interleukin (IL)-28B variant's distribution, insulin resistance, and early virological kinetic changes during antiviral treatment. Relatively few studies have investigated treatment response in prisoners. Reported SVR rates vary widely from 46% to 66% [16–18, 27]. In the present study, we demonstrated a more favorable treatment response in prisoners than in community patients. Several previous reports have also demonstrated a comparable or more favorable treatment response among prisoners than in their counterparts in the community [4, 17, 19, 28]. A unique HCV genotype distribution could partially explain this phenomenon. However, when we evaluated the group infected with HCV genotypes non-1, the treatment success rate was still significantly higher among incarcerated patients. Nevertheless, multivariate analysis revealed that the only two independent predictors of SVR were genotype non-1 and incarceration. Given that medical compliance is a major factor in treatment success, our results could partly be related to poorer patient adherence to therapeutic regimens in community settings. The SVR rate of our non-incarcerated patients was relative lower compared to previous reports in Taiwan. Poor patient adherence to therapeutic regimes might partially explain this result. Eight patients were lost after treatment completion and their SVR information could not be obtained. These patients were all coded as treatment failures. In previous trials, 10–31% of patients discontinued anti-viral treatment because of physical and psychiatric adverse effects [29, 30]. In our study, four community patients discontinued anti-viral therapy because of adverse effects. HCV infection treatment in prison has several unique features, such as closer monitoring of adherence to treatment, management of side effects, and provision of psychiatric care. Thus, treatment in prisons may be associated with higher compliance and consequently with a higher success rate. In the present study, we observed that the number of community patients with HCV infection who were intolerant to the treatment or lost to follow-up assessment of SVR was 12. Conversely, only two incarcerated patients were lost to SVR assessment and no incarcerated patient was intolerant to the treatment. In addition, multiple sessions of therapeutic education were conducted by nurses before and during treatment in our study, further improving the awareness and compliance of the incarcerated patients with HCV infection.

Several new direct antiviral agents (DAAs) have been developed, but many of these drugs are available in only a few countries. Furthermore, the associated high economic burden may preclude their use in poorer regions. This limitation is also apparent in incarcerated institutions, at least in some countries. The rate of reinfection after successful treatment in prisoners is high, especially in IDUs [31]. In our study, four incarcerated patients had virological relapse after completion of treatment. HCV reinfection might account for the reappearance of virus. But in our study population, all enrolled incarcerated patients had completed the treatment course (including the timepoint of SVR) in the prison In theory, they should not have the chance of injecting drugs. The results of previous trails regarding the efficacy and cost-effectiveness of treating incarcerated patients with HCV infection are controversial [32–34], particularly regarding the risk of reinfection in IDUs. Therefore, treatment regimens for patients with HCV infection must account for these barriers. Our study showed a relatively high SVR rate among patients who received peg-IFN-based therapy in an incarcerated setting. This result may help physicians in further treatment decision-making. According to our results, treatment of prisoners with HCV infection is feasible and effective. In addition, our findings highlight the importance of early HCV eradication in the correctional system, because prisons can serve as reservoirs of the amplification of HCV transmission in the community after prisoners are released. Furthermore, we suggest that using periods of incarceration as an opportunity to treat HCV infection is reasonable.

This study had several limitations. First, this study was performed using a retrospective chart review. Second, all male patients treated in this period were enrolled for analyzing treatment response; therefore, the medication adjustments, data collection, and control factors were not standardized. Third, several factors could be associated with SVR rate, such as hepatic fibrosis stage, HIV coinfection, IL-28B variant, and reinfection. However, we did not examine hepatic fibrosis status in all study populations. Fourth, the information regarding the proportion of reinfection and HIV coinfection (in community group) was not complete. Fifth, we previously observed higher favorable IL-28B variant among patients with HCV infection in our hospital [35], but we did not check the IL-28B distribution in the present study. Sixth, because we were unable to evaluate the entire populations of the two selected prisons, the true prevalence of HCV infection among these prisoner populations remained unclear. Seventh, although all included patients were drug dependent, we were unable to collect data on the duration and nature of their drug use behavior. Moreover, our study did not include an assessment of the cost of anti-viral treatment; therefore, the cost-effectiveness analysis could not be performed. Finally, the era of IFN-free DAAs appears to be on the horizon; the peg-IFN-based therapy observed in this study may not remain the mainstream modality of HCV infection treatment.

## Conclusion

This study focused on incarcerated patients with HCV infection and a history of drug dependency. A unique HCV genotype distribution and more favorable treatment response were observed in comparison with community patients with HCV infection. Poorer therapeutic adherence among community patients might partially explain these results. The correctional system may present an optimal opportunity to treat patients with HCV infection and further reduce the public health burden of liver-related complications.
